# Supplementary material for: Understanding Mental Health Needs and Gathering Feedback on Transcutaneous Auricular Vagus Nerve Stimulation as a Potential PTSD Treatment among 9/11 Responders Living with PTSD Symptoms 20 Years Later: A Qualitative Approach
Source: Int J Environ Res Public Health. 2022 Apr 16;19(8):4847. doi: 10.3390/ijerph19084847 (PMC9029393; doi:10.3390/ijerph19084847)
Supplement: Supplementary file 1 [file ijerph-19-04847-s001.zip › ijerph-1594842-supplementary.pdf]

<u>Responder Focus Group Discussion Guide</u>

Focus Group Discussion Guide

Pre-Discussion Orientation

NOTE: Facilitators introduce themselves and describe what a focus group discussion is and how it works. Tell respondents that the focus group discussion will last approximately 90 minutes. Acknowledge the discussion will be audiotape-recorded and provide assurances that all information that they share with us will be kept private. Ensure that <u>all</u> participants understand and have provided consent to the audio recording of the discussion.

Self-Introductions and Warm up

1. Beforehand include one page description of study goals, brief summary of the device and copy of the list of measures.

2. What is a focus group discussion?

3. How do focus group discussions work?

    - interested in your viewpoint, you represent other individuals with PTSD and who may have views just like you
    - research project, not selling anything, just want your perceptions
    - no right or wrong answers
    - honest answers
    - if a particular question or questions make you uncomfortable, you don't have to provide an answer – just ask me to move on to the next question

4. Group defines/outlines rules for mutual respect

    - One person talk at a time
    - Put cell phones on vibrate
    - No judgment
    - Confidentiality of statements shared within group

5. Microphones, recording, assurance of privacy

Introduction

Moderator: Thank you for coming to speak with us today. My name is [XX] and this is [XX]. Today we will be talking generally about mental health care needs of WTC 9/11 responders and your perceptions of what might facilitate or be barriers to engagement in mental health care among 9/11 responders. We will mostly be discussing a possibly new treatment for PTSD called non-invasive transcutaneous auricular vagal nerve stimulation-also known as taVNS. We are looking to provide taVNS to World Trade Center (WTC) responders in order to help with symptoms of Post-Traumatic Stress Disorder (PTSD). We would really like your input on taVNS and how we plan to do our study so that we know

how to adapt it for the specific needs of WTC responders dealing with PTSD. We are very interested in your opinions and there are no right or wrong answers. I want to remind you all at this point that you will remain anonymous for theduration of the study.

There are **three** ways that you can contribute to today's discussion:

1. You can share your personal experience (again, we remind you that what is said here today will remain confidential and anything you tell me won't be linked back to you).

2. You can also share your thoughts and ideas as they pertain to other people with PTSD. For instance, you can talk about someone that you know or that you have heard about (use only pseudonyms).

3. You can share your thoughts about any adjustments that need to be made to the design of the treatment and research study so that it is most relevant and appropriate for use by the target patient population.

These comments will help us to understand how to better help other people like you to improve their quality of life. Thank you again for allowing us the opportunity to speak with you all today. Remember today that you are the expert and we are the students!

ICE BREAKER – What would you be doing now if you weren't with us?

Do you have any questions about the study or the interview before we started?

May we turn on the tape recorder now?

Brief discussion of WTC responder mental health needs.

1. Can you please describe what you see as WTC responders' greatest needs currently as they related to mental health

- Probe for beliefs about mental health care
  - Can mental health care be effective for responders?
  - Who would benefit from mental health care services?

2. Please describe factors that might be barriers for a responder to engage in mental health care

- Transportation
- Work schedule conflicts
- Physical health difficulties
- Occupational barriers
- Fear of repercussions
- Concerns about confidentiality
- Stigma

3. What factors might make it easier for responders to engage in mental health care?
    - WTCHP- access to care/no financial costs
    - Support from job
    - Understanding of mental health risks
    - Support from family/friends

Thank you so much for sharing this with us. We now want to switch gears to discuss a device that might be helpful in reducing PTSD symptoms among those who are suffering who may be in mental health care but are still experiencing symptoms or for those who are not seeking care.

Demonstration of the taVNS device and discussion of the usage Nesos System. Now we are going to take some time to discuss the VNS device and demonstrate how you would use it as if you were a potential participant. Researchers from the Feinstein Institute for Medical Research willdo the demonstration and answer any questions regarding the device

1. First, let us ask: What are your initial impressions of the device itself?
    - Does the device remind you of anything you've used before? Positive or negative impressions
    - Is there anything about the device that makes you uncomfortable?
    - Do you think it will be easy/hard to use the device?
        - PROBE: size of device; ease of transporting it if necessary
    - Do you think it is clear how to use?
    - Any suggestions on language to use?
    - Any issues around having the phone to interface with the device
    -
2. Regarding the device being fitted at the Feinstein Institutes for Medical Research at 350 Community Drive Manhasset, NY
    - Do you think it is reasonable to make the trip in for the fitting? What would be a reasonable time to travel for a fitting?
    - How is the location?

- How do you feel about the device being personally fitted (meaning with your ears being molded and having your own earpiece fitted) to each person?

3. About the time commitment usage with the device. *(Once a day every day for 15 minutes each time over 8 weeks)*
    - Do you think others will use as directed 15 minutes 1x a day?
    - What are some possible obstacles that you can foresee to this?
    - How can we help busy participants fit the sessions into their schedule?
    - How do you think the instructions can be made clearer?
    - What do you think of the overall 8 week window?
    - When do you think a participant might use this (e.g., in the morning before work, at night before bed)?

4. What are some challenges to receiving mental health care among WTC responders?
    - Do you think this will be beneficial for individuals dealing with PTSD?
    - Given the challenges we mentioned, could this help address those issues?
    - Any additional hurdles to using this you can foresee?

Methodology of the study. Thank you for your input. Now we would like to ask some questions about the study protocol so that we ensure that it's acceptable and feasible and that we address potential difficulties with its use and implementation. These questions will help us identify how we might need to modify the intervention (i.e., the administration of the taVNS device) and its delivery so that it is appropriate for the first responders' community.

5. Screening process includes
    - completing a CAPS interview
    - the standard MINI assessment

These additional assessments will be conducted with the responder at either the Northwell CCE or the Feinstein Institute for Medical Research (FIMR), whichever is more convenient for the participant.

- How do you think participants will feel about completing these additional assessments?
- How do you think they will feel about the additional time?
- Do these two locations offer viable options to attend?
- Do you think there should be additional exclusions?
- Other thoughts about increasing ease of screening process for participants?

6. How do you think that participants would respond to a randomization *(2:1)*, meaning that 20 people would receive the intervention and 10 people wouldn't (although all would be compensated for their time completing measures at beginning and end)?

7. Biological assessments and Blood Draws (heart rate monitor, EEG, pupil dilation, galvanic skin response, respiratory rate, blood pressure, facial and neck electromyography)
    - How would you feel about having the following assessments done?
    - How would you feel about having blood drawn?
    - Any suggestions on ways to ease participants' potential concerns?
    - Thoughts about participant compensation for time/transportation?

8. How else can we measure whether people are really benefitting from this program? Please look at this list of measures that we're going to be asking people to complete atthe beginning and end of the 8 weeks. (Rebecca will discuss what each measure is assessing) Please provide your feedback.
    - How do you think the ease of use with a tablet will be?
    - How long do you think it will take to complete these?
    - Are there any topics that aren't covered, but should be?
    - Are there any topics that are being measured that should not be?
    - Any items you think important to include?
    - Reimbursement for time/travel

9. Reimbursement for time/travel
    - $20 reimbursement for the CAPS/MINI regardless of eligibility
    - $25 for baseline measures and bloodwork
    - $25 for follow-up measures and bloodwork
    - All transportation will be reimbursed as well
    - Participants will receive their incentives and travel
    - reimbursement on a ClinCard which functions like a gift card
    - Do you think the reimbursement is adequate given the time?
        - Screening?
        - Baseline & follow-up?
        - Travel costs?

10. Any general feedback including any challenges you see for implementation?

Final comments or suggestions
We've reached the end of the discussion. Do you have anything else that you would like to add about the topics that we have discussed here today?

THANK YOU FOR YOUR TIME AND FOR YOUR IMPORTANT INPUT
